# ANTIDIABETIC THERAPIES METFORMIN AND TRODUSQUEMINE MODULATE MIRNAS INVOLVED WITH LIVER METABOLISM

**DOI:** 10.1093/geroni/igad104.1813

**Published:** 2023-12-21

**Authors:** Sarah Ashiqueali, Augusto Schneider, Xiang Zhu, Ewelena Juszczyk, Yun Zhu, Andrzej Bartke, Shadab Siddiqi, Michal Masternak

**Affiliations:** University of Central Florida College of Medicine, Orlando, Florida, United States; Universidade Federal de Pelotas, Pelotas, Rio Grande do Sul, Brazil; University of Central Florida, Orlando, Florida, United States; Celon Pahrma S.A., Kazuń Nowy, Mazowieckie, Poland; Southern Illinois University, Carbondale, Illinois, United States; Southern Illinois University, Carbondale, Illinois, United States; University of Central Florida College of Medicine, Orlando, Florida, United States; University of Central Florida College of Medicine, Orlando, Florida, United States

## Abstract

Type II diabetes mellitus (T2DM) afflicts approximately 33% of individuals over the age of 65 increasing susceptibility to dyslipidemia, a hallmark of non-alcoholic fatty liver disease (NAFLD). Due to the idiopathic nature of NAFLD, there is no standard of care with only lifestyle intervention strategies available to improve hepatic function. Although dietary modifications and physical activity can prevent the pathogenesis of NAFLD, poor compliance has sparked an urgent need for pharmacological options to achieve glycemic control and prevent the onset of comorbidities. Metformin (MF) is commonly used medication to treat T2DM and has recently gained attention as an anti-aging intervention in non-diabetic individuals. Trodusquemine (MSI-1436) is a drug that induces fat loss by inhibiting the protein tyrosine phosphatase 1B (PTP1B), an enzyme that impairs leptin and insulin signaling. Using miRNA sequencing, we identified a cohort of miRNAs that are influenced by MF and MSI-1436 in the livers of UMHET3 mice. Our in-depth analysis and RT-PCR validation indicated groups of miRNAs regulating the processes of de novo lipogenesis, fatty acid oxidation, very-low-density-lipoprotein and cholesterol transport. Importantly, miR-379-5p, a known modulator of cholesterol metabolism, reported to be inhibited in the livers of patients with NAFLD and in mouse models of diabetes, was found to be upregulated by MSI-1436 in both sexes when comparing with both CTL and MF groups. In summary, our study showed that MSI-1436 more potently inhibits mechanisms involved with liver homeostasis relative to MF yielding insight on its therapeutic potential in concurrent liver diseases resulting from T2DM.

